# Case Report: A Near Miss of Pulmonary Embolism in a Division 1 Collegiate Basketball Player

**DOI:** 10.5811/cpcem.2020.7.47887

**Published:** 2020-09-17

**Authors:** Nicholas M. Chill, Aaron J. Monseau, Brenden J. Balcik, Rosanna D. Sikora, Kathryn Oppenlander

**Affiliations:** West Virginia University School of Medicine, Department of Emergency Medicine, Morgantown, West Virginia

**Keywords:** pulmonary embolism, athlete, PERC

## Abstract

**Introduction:**

The clinical presentation of pulmonary embolism (PE) is often associated with classic vital instability such as tachycardia, hypoxia, and tachypnea. This critical diagnosis is often less likely if a patient is negative by Pulmonary Embolism Rule-Out Criteria (PERC) standards with a low pre-test probability of disease. Caution must be used when evaluating elite athletes with the PERC rule due to low resting heart rate and certain risk factors, which are inherent to athletics.

**Case Report:**

We report the case of a 20-year-old male Division 1 collegiate athlete with pleuritic chest pain diagnosed with PE despite being PERC negative. His presenting heart rate (HR) of 79 beats per minute was correctly determined to be tachycardic relative to his resting HR of 47–60 beats per minute. Despite his PERC negative status, PE was found after an elevated D-dimer and subsequent computed tomography angiography.

**Conclusion:**

Special consideration should be used when evaluating elite athletes for PE, as their resting physiology may differ from the general population. Additionally, certain risk factors for thromboembolic disease are inherent in competitive athletics and should be considered during an initial risk assessment. The presented patient was successfully treated with oral anticoagulation for three months and was able to return to play.

## INTRODUCTION

Pulmonary embolism (PE) is a leading cause of morbidity and mortality worldwide with an estimated 60,000–100,000 deaths per year. It is often associated with chronic and acute medical conditions, recent surgeries, malignancy, obesity, and known thrombophilia.^1^,^2^ A well-conditioned athlete may not fit this classic image, but the nature of competitive athletics exposes them to certain thrombogenic risk factors. With a wide range of presenting symptoms and severity, PE can be a difficult clinical diagnosis. Risk stratification is the initial step in the evaluation for a PE and can be performed using clinical decision rules such as the Wells Criteria or clinician gestalt.^3^

Once the patient is determined to be low risk for PE, the Pulmonary Embolism Rule-Out Criteria (PERC) rule is an effective tool for helping physicians determine the need for further workup and imaging in cases where PE may be considered. The PERC rule is meant to be applied to patients already determined to be low risk for PE and consists of the following eight, easily obtainable, objective measurements and historical factors: age <50; heart rate <100 beats per minute; pulse oximetry >94% at room air; no use of exogenous estrogen; no prior history of venous thromboembolism (VTE); no recent surgery or trauma requiring intubation or hospitalization in the prior four weeks; no unilateral leg swelling.^4^ The PERC rule is 97% sensitive when used appropriately, and can decrease unnecessary testing and ionizing radiation.^4^,^5^ Caution must be used when applying these criteria to elite athletes whose physiology may differ from that of the general population. We discuss the case of a PERC negative, otherwise healthy athlete who was diagnosed with PE, as well as some common diagnostic pitfalls in this patient population.

## CASE REPORT

A 20-year-old Black male, Division 1 collegiate basketball player presented to a student health urgent care clinic with an insidious two-day history of complaint of dry cough, sore throat, and myalgias, as well as chest pain starting one day prior to evaluation. He described his chest pain as an “achy pressure,” radiating down his right arm, intermittent and worse with deep inspiration and laying supine. He denied smoking cigarettes or marijuana but did vape frequently. He had no recent injury, chest exercises, or concern for musculoskeletal injury, or history of deep vein thrombosis (DVT) or PE. The most recent airplane flight was approximately three hours long and greater than six weeks prior to presentation. He had no leg swelling. He had a remote history of right knee arthroscopic meniscus repair seven months prior. He had a history of acid reflux controlled through diet. His vital signs were normal (pulse 79 beats per minute, blood pressure 111/88 millimeters of mercury, respiratory rate 15 breaths per minute, temperature 36.8° Celsius, oxygen saturation 99%). Due to concern for cardiopulmonary pathology, he was sent to the emergency department (ED) for further evaluation.

On arrival to the ED, his vital signs were again normal. He appeared acutely ill and in mild distress. There was no edema to the lower extremities and he did not report leg pain to the emergency providers. His physical exam was otherwise unremarkable. Complete blood count, basic metabolic panel, and troponin were found to be within normal limits. Additional lab work included elevated D-dimer of 592 nanograms (ng) per milliliter (mL) (normal <232 ng/mL). A D-dimer was ordered because of the patient’s status as an athlete. PERC may not have applied due to concern that his presenting heart rate may have been above his baseline. Electrocardiogram and plain chest radiography at the time were normal. A point-of-care echocardiogram showed no evidence of right heart strain and no pericardial effusion.

Given his concerning presentation and abnormal lab work, computed tomography (CT) angiography of the chest was performed demonstrating PE involving the right posterior basal segmental pulmonary artery with associated hemorrhage vs developing pulmonary infarct (Image). He was started on heparin infusion and admitted for further evaluation. After diagnosis of PE was made, the admitting hospitalist noted that there was tenderness along his posterior knee and distal thigh, as well as pain with active and passive range of motion of his knee. These exam findings were not noted in the initial provider’s documentation.

CPC-EM Capsule

What do we already know about this clinical entity?

*Pulmonary embolism (PE) is a critical diagnosis with high morbidity and mortality. Clinical decision tools are available to reliably assess pretest probability*.

What makes this presentation of disease reportable?

*Based off of history and physical exam, the presented patient may fall into the low-risk category for PE*.

What is the major learning point?

*Heart rate is factored into clinical decision tools for PE, but this may be an unreliable metric when evaluating an elite athlete*.

How might this improve emergency medicine practice?

*Knowing inherent risk factors within competitive athletics and understanding athlete physiology can help raise a provider’s suspicion for PE*.

During his hospital course he underwent venous duplex imaging, which was negative for lower extremity DVT, and transthoracic echocardiogram was normal. His oxygen saturations remained above 97% and his highest documented heart rate was 79 beats per minute. His hypercoagulability workup was negative except for equivocal lupus anticoagulant. No clear provoking factors were identified, as his surgery was over six months prior to presentation, and he had no recent team flights or travel in the prior one month. His chest pain and shortness of breath improved on day two of his hospital stay. He was transitioned to apixaban for anticoagulation and discharged home with precautions not to participate in contact sports.

At his three-month follow-up with hematology/oncology, he was found to have a persistently elevated D-dimer and underwent repeat CT angiography of his chest. The CT showed focal right lower lobe opacity consistent with scarring but no residual PE. He was no longer experiencing chest pain or shortness of breath. Additional hypercoagulability workup including protein C, S, and cardiolipin antibody, and beta-2 glycoprotein was negative as well. The PE was ultimately determined to be provoked from either his airplane travel six weeks prior or his arthroscopic meniscus repair seven months prior. He was taken off of anticoagulation and cleared for participation in contact sports. He was instructed to take 81 milligrams aspirin on days of team travel if on a bus or plane. He was able to make a full recovery and returned to competitive Division I basketball.

## DISCUSSION

PE is a critical, potentially life-threatening diagnosis in which a failure to identify may lead to catastrophic morbidity or mortality. Although athletes are typically thought to be lower risk for thromboembolic disease given their elevated activity levels and perception of peak physical fitness, they are often exposed to thrombogenic risk factors that have been shown to make them more susceptible.^6^ Virchow’s triad classically reduces VTE risk factors into three broad categories: hypercoagulability; endothelial damage; and venous stasis.^7^ Athletes are subject to risk factors in all three categories.

Extensive travel and limb immobilization secondary to injury can result in hemostasis. Dehydration and subsequent hemoconcentration following extensive physical exertion can lead to hypercoagulability. Musculoskeletal trauma and surgical intervention from injuries can cause endothelial damage. 6,7 Additionally, Paget-Schroetter syndrome is a cause of effort-related thrombosis in athletes with repetitive overhead motion. With this condition, external rotation of the upper extremity can cause subclavian vein compression between the clavicle and first rib, leading to microtrauma of the endothelium, as well as stasis.^8^,^9^ A combination of the aforementioned circumstances contributed to a total of 55 cases of VTE within professional sports in the United States between 1999–2016, with PE present in 15 of those.^10^ In the case of the presented athlete, PE was suspected to have been provoked from either his airplane travel six weeks prior or his meniscus repair seven months prior.

When evaluating the general population, the PERC has shown to be a valuable tool in reducing unnecessary imaging and ionizing radiation.^5^ However, caution must be used when applying these criteria to elite athletes who have been shown to have lower-than-average resting heart rates.^11^ This caution should be extended to any patient with possible lower resting heart rates from other causes. For example, a patient on a beta blocker may have a blunted tachycardic response to a PE.^12^ It is well known that sinus tachycardia is one of the most common presenting signs in PE. This is reflected in the PERC criteria, which uses greater than 99 beats per minute as a cutoff. The presented athlete’s resting heart rate ranged from 47–60 beats per minute during hospitalization and at outpatient follow-up appointments. His presenting heart rate of 79 beats per minute was an outlier and was correctly interpreted as relative tachycardia. When analyzing this patient using PERC criteria, his total score was zero, which precluded any further testing for PE.

Had the attending physician not recognized relative tachycardia, deemed this patient as moderate risk and subsequently ordered D-dimer for further evaluation, the diagnosis of PE might have been missed given his otherwise negative workup. An argument can be made that since he did not report any symptoms of DVT initially he could have been deemed low risk and the PERC rule could have been applied. He would have been PERC negative, a D-dimer would not have been ordered, and the diagnosis of PE would have been missed. While the Wells Criteria score is often used as an objective measure for risk stratification in PE, it is also prone to error in a patient population that may not have a normal physiologic response when compared to the general population, such as relative tachycardia. This underscores the importance of understanding these clinical decision rules as well as the patient’s baseline physiology.

While the PERC rule is often praised for its ease of use with objective measurements, it must be restated that it should only be applied to a low-risk population. It is up to the provider to determine whether the patient falls into the low-risk population, which remains a largely subjective decision using the Wells Criteria or clinician gestalt. With the classic association between VTE and inactivity, obesity, and chronic medical conditions, athletes may be at risk of a missed diagnosis, as they are often perceived as lowest risk due to their cardiovascular health and fitness.

## CONCLUSION

Pulmonary embolism can be an elusive diagnosis but should be considered frequently due to the potential for significant morbidity and mortality. It is a misconception that a healthy, well-appearing athlete is devoid of risk factors for VTE, as the inherent nature of competitive athletics exposes individuals to conditions that may promote thrombosis. Additionally, vital signs that may appear normal for a typical patient may be abnormal in an athlete with high physiologic reserve. Clinical decision rules are excellent tools for ruling out PE when used appropriately, but some pitfalls remain when evaluating elite athletes.

## Figures and Tables

**Image:**
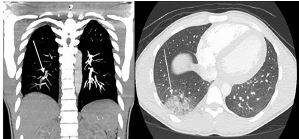
Computed tomographic angiography of the chest in coronal (left) and axial (right) views. Arrows point to pulmonary embolism involving the right posterior basal segmental pulmonary artery (left) and associated hemorrhage versus developing pulmonary infarct (right).
